# Supplementary material for: Atrial fibrillation in middle-aged athletes: Impact on left atrial, ventricular and exercise performance
Source: PLoS One. 2024 Mar 13;19(3):e0294367. doi: 10.1371/journal.pone.0294367 (PMC10936837; doi:10.1371/journal.pone.0294367)
Supplement: S1 Table — Data are mean ± SD (range). LAMAX; Left atrial maximal volume, LAMIN; left atrial minimal volume, LAPRE-A; left atrial pre-contractile volume, LARES; left atrial reservoir volume, LAPASS; left atrial passive emptying volume, LACOND; left atrial conduit volume, LABOOST; left atrial booster volume. (DOCX) [file pone.0294367.s001.docx]

**S1 Table. Absolute LA volumes and phasic function**

| **Variable** | **Rest** | | |  | **Light** | |  | **Moderate** | |  |
| --- | --- | --- | --- | --- | --- | --- | --- | --- | --- | --- |
|  | EA | EA-AF | | *p* | EA | EA-AF | *p* | EA | EA-AF | *p* |
| **Absolute** **(mL)** | | | | | | | | | | |
| LA_MAX_ | 48 ± 12  (31-67) | 52 ± 11  (34-70) | *0.503* | | 64 ± 10  (39-87) | 59 ± 17  (41-90) | *0.382* | 59 ± 8  (42-76) | 60 ± 15  (38-79) | *0.573* |
| LA_MIN_ | 15 ± 5  (9-26) | 17 ± 7  (12-29) | *0.605* | | 20 ± 9  (10-45) | 19 ± 9  (9-37) | *0.907* | 17 ± 6  (8-32) | 20 ± 9  (5-32) | *0.406* |
| LA_PRE-A_ | 29 ± 8  (19-44) | 30 ± 9  (20-44) | *0.894* | | 49 ± 11  (32-65) | 38 ± 14  (22-61) | *0.039* | 58 ± 7  (42-67) | 52 ± 16  (27-76) | *0.232* |
| LA_RES_ | 33 ± 9  (21-46) | 35 ± 7  (23-48) | *0.558* | | 44 ± 5  (28-51) | 40 ± 10  (25-62) | *0.169* | 42 ± 6  (28-51) | 37 ± 8  (27-50) | *0.079* |
| LA_PASS_ | 20 ± 5  (11-27) | 22 ± 7  (8-33) | *0.407* | | 15 ± 12  (0-31) | 21 ± 11  (6-38) | *0.218* | 1 ± 3  (0-10) | 5 ± 5  (0-15) | *0.090* |
| LA_COND_ | 55 ±12  (34-71) | 42 ± 13  (27-65) | ***0.043*** | | 68 ± 11  (50-84) | 57 ± 10  (47-74) | ***0.037*** | 81 ± 12  (55-95) | 59 ± 11  (40-84) | ***0.001*** |
| LA_BOOST_ | 13 ± 5  (4-20) | 13 ± 4  (6-20) | *0.737* | | 29 ± 13  (10-49) | 19 ± 8  (7-34) | ***0.033*** | 41 ± 6  (28-51) | 32 ± 10  (13-48) | ***0.020*** |
|  |  |  | |  |  |  |  |  |  |  |

Data are mean ± SD (range). LA_MAX_; Left atrial maximal volume, LA_MIN_; left atrial minimal volume, LA_PRE-A_; left atrial pre-contractile volume, LA_RES_; left atrial reservoir volume, LA_PASS_; left atrial passive emptying volume, LA_COND_; left atrial conduit volume, LA_BOOST_; left atrial booster volume.
