# Supplementary material for: Atrial fibrillation in middle-aged athletes: Impact on left atrial, ventricular and exercise performance
Source: PLoS One. 2024 Mar 13;19(3):e0294367. doi: 10.1371/journal.pone.0294367 (PMC10936837; doi:10.1371/journal.pone.0294367)
Supplement: Attachment — Submitted filename: Response to Reviewers.docx [file pone.0294367.s002.docx]

**Response to Reviewers**

**Reviewer #1:**

1. *The presence of paroxysmal atrial fibrillation in athletes has previously been verified by clinical diagnosis using Holter or loop ECG. However, it is not described whether the presence of asymptomatic AF was tested in the control group, for example with ECG Holter. if it has not been done, it must be indicated in the methods and represents a limitation of the study.*

We appreciate the reviewer’s comment. Healthy endurance athletes (EA) underwent medical screening for this reason and the methods section has been revised to include the following:

**“Healthy athlete recruits underwent medical screening including a full medical history, resting ECG and 48 hr Holter monitor.”**

While we cannot rule out AF with 100% certainty, the absence of any symptomatic history and ECG/Holter evidence of arrhythmias reflects a rigorous effort to ensure a very low likelihood of AF or cardiovascular disease in this cohort. We included this statement in the results:

**“Healthy athletes were free of medication or history of premature atrial contractions or symptoms at rest or during exercise, and had no evidence of arrhythmias on resting ECG or 48 hour Holter monitoring.”**

2. *Four athletes with AF were treated with negative inotropic drugs and this may have influenced the value of ventricular stroke volume during exercise. It would be useful to evaluate the data only from athletes without therapy and discuss this aspect, also including it in the limitations of the study.*

This is a valid concern. Our protocol used a target heart rate (HR) approach at both levels of exercise. We examined the individual response of patients on rate-control / negative inotropic medication (only 4 participants) and the presence of medication *did not* impede their ability to reach the HR targets. There is also some evidence that Beta blockers do not affect stroke volume at any given heart rate with training. (Mier CM, Domenick MA, Wilmore JH. Changes in stroke volume with beta-blockade before and after 10 days of exercise training in men and women. J Appl Physiol (1985). 1997 Nov;83(5):1660-5.)

We have also included data for mean HR achieved at each exercise stage for both groups of participants in Table 2, and there was no difference between groups. Notwithstanding we have inserted this statement into the exercise response section in the manuscript:

**Results:**

**Added Heading: “Cardiac Function During Exercise”**

**“All participants were able to complete light and exercise protocols, reaching the target HR for both levels of intensity regardless of medication status in the EA-AF group, and there were no AF episodes the day of, or day before exercise assessments”**

**Limitations:**

**“…while EA-AF athletes on negative inotropic medication achieved target heart rates during exercise, it is possible that atrial function was affected.”**

3. *Differences in VO2 peak between the two study groups (p 0,05) is not discussed in the text. Furthermore, it is not indicated how many patients performed a maximal test and therefore VO2 peak was assessed.

   Table 1: EA-AF have a higher BMI, this could be a relevant aspect and deserves a discussion.*

These are valid concerns, and we agree that these findings warrant some elaboration in the manuscript. All participants completed all study protocols including maximal exercise testing. By convention, as with many studies that conduct maximal’ exercise testing, we report the peak value obtained, or “VO_2_peak”, given the low percentage of individuals who demonstrate a true maximal effort as defined by a plateau in oxygen consumption. As for BMI, all participants had BMIs in the normal range, well below levels associated with elevated AF risk. We did not measure fat or lean mass. We are not aware of specific links between these measures and AF risk, nor can we confirm with certainty if the lower *relative* VO_2_peak values (normalized for body mass) in the EA-AF group was due to a higher fat mass and therefore, higher BMI. It is possible that some degree of detraining since AF diagnosis contributed to this finding, yet data from long-term exercise histories remained very similar. These issues are now addressed directly in the Results and Discussion as outline below.

***Results:***

***“*Twenty-nine male endurance athletes completed all study protocols.”**

***Results:***

**“A similar absolute VO_2_peak (L/min) was observed for the EA and EA-AF group, however when normalized for body mass, a lower relative VO_2_peak (mL/kg/min) was observed EA-AF group (p=0.05).”**

***Limitations:***

**“We note that several of the EA-AF participants reported a modest reduction in training intensity following diagnosis of AF which may have contributed to a higher BMI and lower relative VO_2_peak. While some degree of recent detraining may have occurred in the EA-AF group since AF diagnosis, lifetime exercise history and weekly exercise volumes were similar between groups. Given evidence that exercise-induced LV cavity remodelling can persist for years after exercise cessation (35), it is likely that any reduction in cardiac dimensions from attenuated training would be very small .”**

4. Table 3: LA pre-A is significantly lower in EA-AF athletes only in the mild exercise group. Is there a possible explanation for this data?

We appreciate the reviewer’s comment. The LA pre-A volume represents the residual volume in the atrium prior to atrial contraction and following passive flow of blood from the atrium to the left ventricle. Our athletes with AF had a smaller LA pre-A volume suggesting less volume remaining in the atrium for booster function/contractile output, which was reflected in both booster volumes at light and moderate exercise intensities. As included in the Discussion section, it is possible that our athletes with AF have more passive emptying (possibly due to higher diastolic left atrial pressures). These points are integrated into a revised passage within the Discussion:

**“In the present study, EA were able to recruit active atrial emptying at light exercise intensity to a greater extent than the EA-AF group, who in turn had more passive emptying, possibly due to higher diastolic left atrial pressures. It is possible that athletes are more dependent on passive emptying to maintain SV in the absence of booster function, particularly during exercise when episodes of AF occur (26).”**

Page 8: Inter-AEMD was defined as (lateral-tricuspid PA), intra-right as (septal PA-tricuspid PA), and intra-left as (lateral PA-septal PA). There's probably a typo, the brackets are not needed.

**Thank you. This has been corrected.**

5. *Abstract: the AEMD acronym is missing.*

**Thank you. This has been corrected.**

**Reviewer #2:**

1. *Congratulations to the authors for the very interesting idea of the manuscript. The characterization of AF in athletes is an hot topic and deserves numerous studies to be explored.
   However I have many concerns about this study:
   The only difference in baseline characteristics between the two groups is represented by the BMI; for athletes it would be more appropriate to describe the percentage of muscle mass and fat mass, because we know that an elevated BMI, in non athlete population, is associated with an enhanced risk of developing AF, but an elevated BMI in athletes could be addressed to a relevant muscle mass and not to a overweight condition due to fat excess.*

We have provided a response (and edited the manuscript) to address this concern raised by Reviewer 1, above. Despite the higher BMIs in the EA-AF group, the values are well within the normal range for this age-group and are not likely to have clinical relevance. It is possible the slightly higher BMIs are due to some degree of detraining but we did not measure fat or lean mass. The higher BMI likely contributed directly to the slightly lower “relative” VO_2_peak (normalized for body mass), but similar “absolute”, VO_2_peak (L/min). We thank the reviewer for these insights, and trust our changes (see above) address these concerns.

2. *In addition to a critical mass which is necessary as a substrate to initiate AF, atrial cardiomyopathy is required, and all the CHADVACS score components are a determinant of atrial cardiomyopathy; so you should add the CHADVASC Score for the group of participants;*

   Thank you for this important point; we have added the following to our Methods and Results sections:

*Methods:*  **“A CHA_2_DS_2_VASC score was calculated for athletes with AF.”**

*Results:*

**“A total of four athletes with AF were identified to have a CHA_2_DS_2_VASC greater than 0; three athletes had a cumulative score of 1 (due to hypertension), and one athlete had a cumulative score of 2 (due to hypertension and age>65 yrs.).”**

3. *Considering the characteristic of athlete population and their high rate of spontaneous AF cardioversion, you should include a reference about this topic, such as: “Mariani MV, Pierucci N, Piro A, Trivigno S, Chimenti C, Galardo G, Miraldi F, Vizza CD. Incidence and Determinants of Spontaneous Cardioversion of Early Onset Symptomatic Atrial Fibrillation. Medicina (Kaunas). 2022 Oct 24;58(11):1513. doi: 10.3390/medicina58111513.”*

   Thank you for bringing this important paper to our attention. Our AF population was confined to those with paroxysmal AF who had a relatively low AF burden; none were in AF the day of or the day prior to the study.

   We have added the following to the discussion:

**“Our cohort had a relatively low AF burden, including the frequency and duration of AF episodes, which usually spontaneously converts in athletes (Mariani et al).”**

4. *Moreover you should specify if any of them were on antiarrhythmic drugs.*

This information is already present in the Results section, pg. 11 but has been simplified as follows:

**“A total of 5 EA-AF participants were on a beta blocker, anticoagulant or calcium channel blocker, and one was also on an antiarrhythmic. In all cases, medications remained unchanged during all assessments.”**
